# Insulin- like Growth Factor-Binding Protein Action in Bone Tissue: A Key Role for Pregnancy- Associated Plasma Protein-A

**DOI:** 10.3389/fendo.2018.00031

**Published:** 2018-02-16

**Authors:** James Beattie, Hasanain Al-Khafaji, Pernille R. Noer, Hanaa Esa Alkharobi, Aishah Alhodhodi, Josephine Meade, Reem El-Gendy, Claus Oxvig

**Affiliations:** ^1^Division of Oral Biology, Leeds School of Dentistry, Level 7 Wellcome Trust Brenner Building, University of Leeds, St James University Hospital, Leeds, United Kingdom; ^2^Department of Molecular Biology and Genetics, Aarhus University, Aarhus, Denmark; ^3^Department of Oral Biology, Dental College, King AbdulAziz University, Jeddah, Saudi Arabia; ^4^Department of Oral Pathology, Faculty of Dentistry, Suez Canal University, Ismailia, Egypt

**Keywords:** insulin-like growth factor-binding protein-4, bone, pregnancy-associated plasma protein-A, proteolysis, insulin-like growth factor-binding protein-5

## Abstract

The insulin-like growth factor (IGF) axis is required for the differentiation, development, and maintenance of bone tissue. Accordingly, dysregulation of this axis is associated with various skeletal pathologies including growth abnormalities and compromised bone structure. It is becoming increasingly apparent that the action of the IGF axis must be viewed holistically taking into account not just the actions of the growth factors and receptors, but also the influence of soluble high affinity IGF binding proteins (IGFBPs).There is a recognition that IGFBPs exert IGF-dependent and IGF-independent effects in bone and other tissues and that an understanding of the mechanisms of action of IGFBPs and their regulation in the pericellular environment impact critically on tissue physiology. In this respect, a group of IGFBP proteinases (which may be considered as ancillary members of the IGF axis) play a crucial role in regulating IGFBP function. In this model, cleavage of IGFBPs by specific proteinases into fragments with lower affinity for growth factor(s) regulates the partition of IGFs between IGFBPs and cell surface IGF receptors. In this review, we examine the importance of IGFBP function in bone tissue with special emphasis on the role of pregnancy associated plasma protein-A (PAPP-A). We examine the function of PAPP-A primarily as an IGFBP-4 proteinase and present evidence that PAPP-A induced cleavage of IGFBP-4 is potentially a key regulatory step in bone metabolism. We also highlight some recent findings with regard to IGFBP-2 and IGFBP-5 (also PAPP-A substrates) function in bone tissue and briefly discuss the actions of the other three IGFBPs (-1, -3, and -6) in this tissue. Although our main focus will be in bone we will allude to IGFBP activity in other cells and tissues where appropriate.

## Introduction

The insulin-like growth factor (IGF) axis comprises two polypeptide growth factors (IGF1 and IGF2), two cell surface receptors [IGF1 receptor (IGF1R) and IGF2R], and six soluble high-affinity IGF-binding proteins (IGFBP-1–6). Ancillary proteins associated with the IGF axis include various IGFBP proteinases that cleave IGFBPs into fragments with greatly reduced IGF-binding affinity, thus regulating the partition of IGFs between IGFBPs and cell surface receptors (1). IGFs are present at high concentrations in bone matrix (2), and disruption of the IGF1 gene compromises skeletal growth in mice (3) and humans (4). In addition to an anabolic role in mature bone, IGFs also stimulate differentiation of osteoblasts in developing bone tissue and regulate the balance between bone accretion and resorption which occur throughout life (5–7). IGFBP-4 is abundantly expressed in bone tissue (8), and the role of this IGFBP in regulating bone metabolism has been extensively investigated (9–11). In recent years, the activity of a specific IGFBP-4 proteinase, pregnancy-associated plasma protein-A (PAPP-A), has also been investigated in bone and other tissues (12–14). IGFBP-2 and IGFBP-5 are also significantly active in bone tissue demonstrating both IGF-dependent and IGF-independent effects (15–18). Additionally, signaling pathways associated with IGFBP-2 and IGFBP-5 action in osteoblasts and bone tissues have been recently reported (19–22). In this review, we touch on each of these topics and also briefly on the actions of the other IGFBPs (IGFBP-1, -3, and -6) in bone cells and tissues.

## Insulin-Like Growth Factor-Binding Protein-4

Insulin-like growth factor-binding protein-4 was first identified as an inhibitory IGFBP in medium conditioned by the TE89 human osteosarcoma cell line (23) and then cloned from cDNA libraries of various tissues in human and rat (24, 25). It is a 237-residue protein sharing the 3-domain structure previously described for other IGFBPs. Early studies showed that IGFBP-4 inhibited IGF2-stimulated thymidine uptake in primary cultures of human osteoblasts (hOB) (26) and in the MC3T3-E1 mouse osteoblast cell line (27) and inhibited IGF1-stimulated aminoisobutyrate uptake in bovine fibroblasts and in the rat neuronal B104 cell line (28, 29). This inhibitory activity *in vitro* led to the hypothesis that IGFBP-4 generally displayed anti-anabolic and anti-proliferative effects. In confirmation of this, overexpression of IGFBP-4 in a malignant prostate epithelial cell line decreased the proliferative response to IGF1 and delayed tumor development when transfected cells were transplanted into nude mice (30). *In vivo* data also supported an IGF-inhibitory role for IGFBP-4. Tissue-specific overexpression of IGFBP-4 in smooth muscle cells using an α-actin promoter caused smooth muscle hypoplasia (31) and a similar strategy using a protease resistant form of IGFBP-4 (pr IGFBP-4) (see below) resulted in transgenic mice with decreased internal smooth muscle mass in stomach, bladder, and aorta (32). Importantly, with respect to this review, IGFBP-4 overexpression in osteoblasts decreased bone formation and compromised global skeletal growth (11). Some epidemiological data also supported an inhibitory role for IGFBP-4 with increased levels in a cohort of female patients with age-related osteoporotic fractures of the hip and spine (33). Although this evidence suggested an inhibitory role for IGFBP-4, other reports indicated an anabolic role for IGFBP-4. Therefore, systemic administration of IGFBP-4 to mice increased bone tissue markers (osteocalcin and alkaline phosphatase) in serum and skeletal tissues (10). Additionally, IGFBP-4 knockout (KO) mice exhibited prenatal growth retardation, suggesting that IGFBP-4 may be required for full growth promoting effects of IGF2 in the fetus (34). IGFBP-4 KO mice also showed gender dependent changes in skeletal phenotype with female mice having reduced bone mineral density (BMD) along with other features associated with osteopenia (9). Clearly, further research is required to definitively establish the role of IGFBP-4 in bone tissue physiology. In this respect, the observation of IGFBP-4 proteolysis by fibroblast and bone cell cultures has attracted much interest as a means of regulating the activity of IGFs in bone and other tissues, and we provide a short summary of this area in the following section.

## IGFBP-4 Proteolysis

Addition of IGF1 to cultures of human fibroblasts reduced the levels of a 24 kDa IGFBP in conditioned medium and development of specific antibodies confirmed this species as IGFBP4 (35, 36). IGF1-dependent downregulation of IGFBP-4 occurred independently of IGF1R activation and was not associated with changes in IGFBP4 mRNA levels, suggesting a direct post-translational regulation of IGFBP-4. Shortly thereafter, IGF-induced decreases in IGFBP-4 protein levels were shown to be due to the presence of a proteolytic activity in fibroblast-conditioned medium which in cell-free assays was activated by IGF1 or IGF2 (37). IGFBP-4 was cleaved into two discrete fragments by this protease, suggesting a specific cleavage point within the protein (38). The cleavage site was identified at the peptide bond M135-K136 within the central domain of IGFBP-4 producing 14 and 18 kDa protein fragments (29). These data were used to engineer protease-resistant IGFBP-4 mutants that have proven useful in the further study of the biological significance of IGFBP-4 proteolysis (29, 39). This became apparent when intact, but not cleaved IGFBP-4, was shown to inhibit [^3^H] aminoisobutyric acid uptake into bovine fibroblasts with the inference that cleaved IGFBP-4 fragments did not bind IGF1. Further study indicated that IGF2 was a more potent activator of IGFBP-4 cleavage than IGF1 and IGF2 pre-treatment of human dermal fibroblast cultures increased sensitivity of cell cultures to IGF1. The concept of IGF2-mediated IGFBP4 cleavage as a route for increasing sensitivity to IGF1 (40) may be significant as IGF1 and IGF2 are usually present together in the pericellular environment, suggesting a complex interaction between the growth factors to regulate anabolic responses.

Primary cultures of hOB expressed an IGFBP-4 protease activity identical to that described for fibroblasts (41), and pre-treatment of osteoblast cultures with IGF2 also increased sensitivity to IGF1-stimulated [^3^H] thymidine incorporation (42). Subsequently, IGFBP-4 protease activity has been reported in human endometrial stromal cells (43) and in porcine aorta-derived smooth muscle cells (44), suggesting that proteolysis of IGFBP-4 may have widespread biological significance. At around this time, a landmark study identified PAPP-A as the enzyme responsible for IGF-dependent cleavage of IGFBP-4 in fibroblast-conditioned medium (34). PAPP-A was also shown to cleave IGFBP-5 in an IGF-independent manner (45). Identification of PAPP-A as the IGF-dependent IGFBP-4 proteinase caused a paradigm shift in this area of IGF research. Whereas previously IGFBP-4 had been viewed mainly as an IGF-inhibitory IGFBP in tissue culture studies, co-expression of PAPP-A in cell culture could negate this inhibitory effect. Furthermore, the “activation” of PAPP-A by IGFs suggested possible positive feedback loop whereby growth factor action could be enhanced. Further aspects of function, structure, and regulation of PAPP-A activity are discussed below.

## Pregnancy-Associated Plasma Protein-A

### Functional Aspects

Pregnancy-Associated Plasma Protein-A was partially purified from human fibroblast-conditioned medium by Lawrence et al (46), and its identity was confirmed by mass spectroscopy. By using polyclonal anti-PAPP-A antibodies, IGFBP-4 protease activity in fibroblast-conditioned medium could be completely inhibited, suggesting that PAPP-A may be the only IGFBP-4 protease expressed by these cells. PAPP-A isolated from fibroblast cultures was found to be identical to the enzyme described in pregnant serum (46–48), showing both IGF dependency and the same site of proteolytic cleavage in the central domain of IGFBP-4 (see above). Identification of PAPP-A allowed some elegant transgenic studies highlighting the importance of this enzyme. Transgenic mice with a collagen I promoter–PAPP-A construct overexpressed PAPP-A specifically in osteoid tissue causing increased calvarial BMD (14). In double transgenic mice overexpressing PAPP-A and a pr IGFBP-4, bone phenotype was similar to single pr IGFBP-4 transgenics, showing decreased calvarial thickness and BMD compared to WT mice. This provided strong evidence that *in vivo* anabolic effects of PAPP-A were due to IGFBP-4 proteolysis, most likely resulting in an increase in the local bioavailability of IGF (49). In confirmation of this, PAPP-A KO mice showed reduced femur BMD and blunted responses to the anabolic actions of parathyroid hormone (12). In a clinical context, PAPP-A has been proposed as a target for anti-proliferative therapies in various cancers. Studies in an ovarian cancer tissue model (50) and using xenografts of adenocarcinoma A549 cells (45) showed that antibody-mediated inhibition of PAPP-A activity decreased tumor growth presumably because pericellular IGF remains bound to IGFBPs leading to a reduction in free IGF in the local tumor environment. This may be important as current anti-IGF-based strategies have proved disappointing in clinical trials. Anti-IGF1R strategies are hampered by hyperinsulinemia secondary to elevated GH levels as a result of impaired IGF-1 feedback at the level of the pituitary (51). This may lead to increased mitogenic signaling by elevated insulin levels through the insulin receptor (IR). IGF1R blockade may also result in IGF1 signaling through the IR or through hybrid IGF1R/IR isoforms which are known to exist in many tissues (52) and which may not be blocked by anti-IGF1R-directed monoclonal antibodies. See the study by Yee (53) for an excellent review of the abovementioned arguments. In contrast, the use of anti-PAPP-A-directed antibodies would not be associated with these complications acting only to inhibit IGF1 release from pericellularly proteolysed IGFBP:IGF complexes.

### Structural Aspects

Although PAPP-A was isolated over four decades ago from pregnancy serum (54), it was only after the cloning and expression of this large (1,547 residues) protein that detailed work on protein structure began (55). PAPP-A belongs to the metzincin superfamily of metalloendopeptidases containing a Zn-binding motif and a highly structurally conserved Met-turn (56). PAPP-A associates with the cell surface through two of five short consensus repeat modules within the C-terminus of the protein, and membrane-bound PAPP-A remains catalytically active. This may ensure release of IGF in the vicinity of cell surface IGF1R (57). Under reducing conditions, PAPP-A migrates as a 200 kDa protein although in pregnancy serum (and some other biological fluids) it is primarily present as a disulfide-bound dimer associated covalently with another disulfide bound dimer of the proform of eosinophil major basic protein (proMBP) in a 2:2 heterotetrameric complex (58, 59). The structure of the heterotetrameric PAPP-A:proMBP complex identifies a disulfide bridged dimer of PAPP-A covalently bound to a disulfide-bridged dimer of proMBP *via* two interchain disulfide bridges (60). In this configuration, PAPP-A is inactive with the proMBP dimer binding at or close to the active site of PAPP-A, suggesting that steric inhibition of enzyme activity may result. Both PAPP-A and proMBP are extensively glycosylated, and under non-denaturing gel electrophoresis conditions, the complex runs as a large (>500 kDa) molecular weight species. A mutagenic analysis of the substrate IGFBP-4 suggested that the C-terminal domain of IGFBP-4 conferred the IGF dependence for PAPP-A cleavage of IGFBP-4 (61). In addition, this same study showed that the region between the Zn-binding domain and the Met turn motif of PAPP-A was important for proteolytic activity toward the IGFBP-4:IGF1 complex. Availability of recombinant PAPP-A allowed confirmation that the rate of IGFBP-4 proteolysis is enhanced by binding of IGFs to IGFBP-4 (62), and detailed kinetic analysis confirmed IGF2 as a more potent activator of proteolysis than IGF1. The effect of IGFs on IGFBP4 proteolysis was associated with changes in both affinity (*K*_m_) and turnover rate (*K*_cat_). This study also confirmed IGFBP-5 as a PAPP-A substrate although proteolysis of IGFBP5 was not IGF dependent (63). Further mutational analysis suggested that the Lin12-Notch repeat modules present in the C-terminal of PAPP-A are responsible for the differential requirement of IGFBP-4 and IGFBP-5 for IGF during PAPP-A-mediated proteolysis (64, 65).

### Regulation of PAPP-A Activity

Relatively few agents have been shown to influence PAPP-A activity. IGFBP-4 proteolysis was inhibited following treatment of fibroblast cultures with phorbol esters. The attenuation of this effect by prior treatment with actinomycin D or cycloheximide suggested PCK-regulated expression of an inhibitor of IGFBP-4 proteolysis (66). Such an inhibitory activity was also reported in SV40-transformed hOB cells, suggesting that the process of cellular transformation may be associated with inhibition of IGFBP-4 proteolysis (67). The finding that phorbol esters and/or SV40-mediated transformation increased the expression of proMBP – a covalent inhibitor of PAPP-A (see above) – suggested at least one route by which these agents may act to inhibit IGFBP-4 cleavage in fibroblast cultures (68).

An early study reported stimulation of IGFBP-4 proteolytic activity in the rat neuronal B104 cell line by glucocorticoids (69) and following identification of PAPP-A as an IGFBP-4 proteinase, the synthetic glucocorticoid dexamethasone was shown to increase enzyme activity in primary cultures of rat vertebral osteoblasts (13). PAPP-A mRNA levels were not altered by dexamethasone treatment, suggesting a post-transcriptional mechanism by which enzyme activity was increased. In contrast to the above, TGFβ increased PAPP-A mRNA levels approximately 12-fold in hOB cultures, and this was associated with increased PAPP-A activity in conditioned medium (70). The demonstration of increased IGF2-mediated IGFBP-4 cleavage following TGFβ treatment of hOB cultures (71) may be of particular significance given the fact that IGF2 and TGFβ are two of the most abundant growth factors present in bone matrix, and a co-ordinated action of TGFβ and IGF2 in bone matrix to increase local availability of IGF may occur. Osteoblasts secrete IGF peptides endogenously (IGF2 > IGF1) and, despite the fact that the IGFBP-4 levels in osteoblast-conditioned media are typically an order of magnitude higher that the IGF2 levels, endogenous IGF2 can stimulate the proteolysis of concurrently expressed IGFBP-4 protein in osteoblast cultures (72, 73).

**Figure 1:**
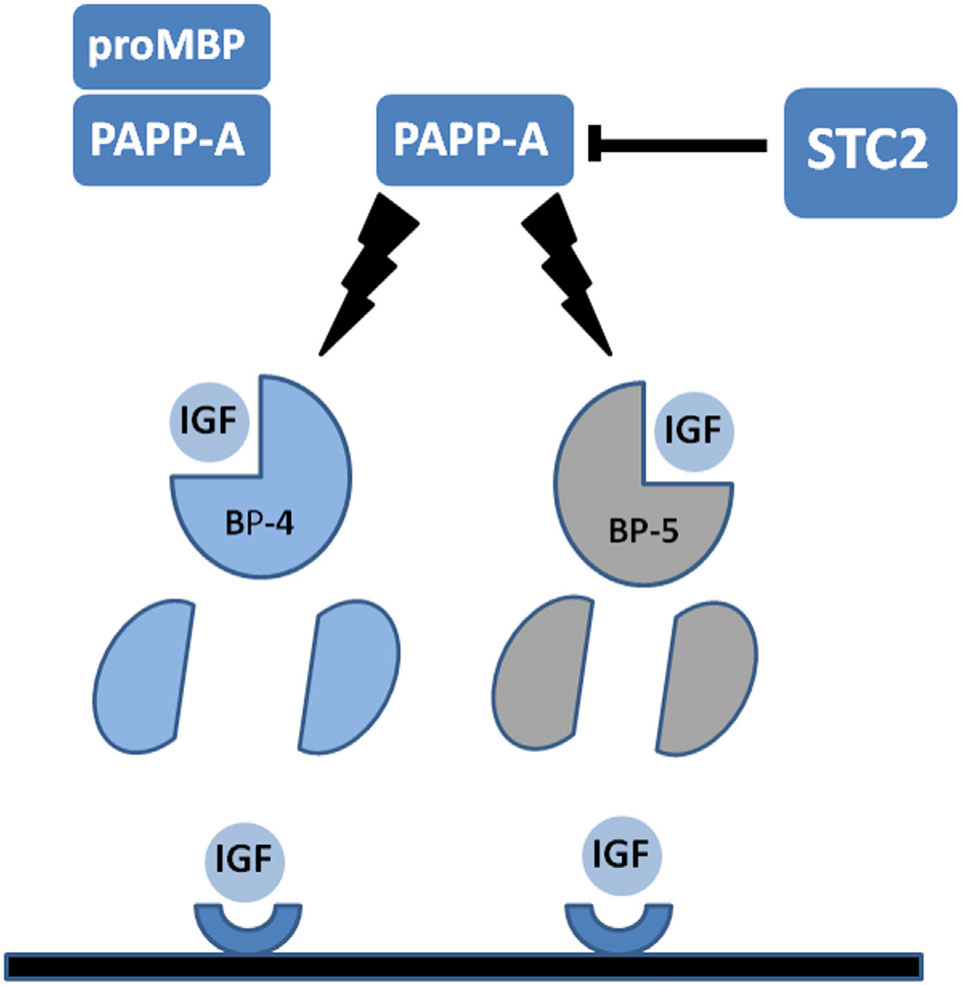
Diagrammatic representation of pregnancy-associated plasma protein-A (PAPP-A) activity in the insulin-like growth factor (IGF) axis. PAPP-A is present in serum and some other biological fluids covalently complexed with the pro-form of eosinophil major basic protein (proMBP). In this form PAPP-A is inactive. Uncomplexed PAPP-A acts to cleave IGF-binding protein-4 (IGFBP-4) and IGFBP-5 into fragments with reduced IGF-binding affinity. IGFBP-4 but not IGFBP-5 requires binding of IGF for PAPP-A cleavage. Proteolysis of IGFBP substrates releases IGFs to allow interaction with cognate cell surface receptors. Recently, discovered stanniocalcins (STC1 and STC2) act to inhibit PAPP-A activity. STC2 is shown, and this inhibitor forms a covalent bond with PAPP-A to inhibit the proteolytic activity. See the text for further details.

Recently, two novel protein inhibitors of PAPP-A activity have been described. These are members of the stanniocalcin family (STC1 and STC2) and were first identified as regulators of Ca homeostasis in teleost fish (74, 75). However, in the context of the mammalian IGF axis, their status as PAPP-A inhibitors indicates that these proteins are negative growth regulators. Overexpression of STC1 or STC2 resulted in growth retardation in transgenic mice (76, 77), whereas KO of STC2 causes increased growth (78). Molecular mechanisms of STC1 and STC2 inhibition of PAPP-A differ with STC2 forming a disulfide-bonded covalent complex with PAPP-A and STC1 forming a high-affinity non-covalent complex with the enzyme. Nonetheless, both STC1 and STC2 potently inhibit PAPP-A which may cause an increased concentration of IGF bound in complex with IGFBP-4 (and IGFBP-5, see below) and hence less bioavailable IGF in the pericellular environment. In agreement with this, STC2 inhibited PAPP-A-stimulated IGF1R phosphorylation in transfected cells exposed to IGF1:IGFBP-4 complexes (74). A recent study using whole exome sequencing of a large human cohort reported two separate single amino acid mutations of STC2 leading to compromised inhibition of PAPP-A. The fact that these alleles strongly associated with increased height in the sampled population is of particular interest (79). A diagrammatic representation of the IGFBP-PAPP-A-STC axis is presented in Figure 1.

## PAPP-A in Other Species

Pregnancy-associated plasma protein-A has also been cloned from a mouse cDNA library (80). Although murine (m) PAPP-A shares 91% homology with the human enzyme and cleaves IGFBP-4 in an IGF-dependent manner, mPAPP-A activity is not elevated in pregnant serum or in placenta. In addition, a variant mPAPP-A containing a 29-residue insert (PAPP-Ai) was also isolated. Interestingly, this PAPP-A isoform was a less efficient IGFBP-4 protease than the shorter variant of the enzyme. The significance of these differences between murine and human PAPP-A remains to be resolved, although PAPP-A null mice are 40% smaller than littermates, suggesting a role for PAPP-A during embryogenesis (81). This may be due to diminished IGFBP-4 cleavage and, therefore, reduced IGF availability in the developing fetus. In agreement with this, IGFBP-4 cleavage is absent in fibroblast cultures derived from these null mice. PAPP-A is present in multiple other species, including zebrafish, and interestingly, the absence of PAPP-A in this species causes a developmental delay, which is independent of proteolytic activity (82).

## Pregnancy-Associated Plasma Protein-A2

Overgaard et al. described the cloning of a metalloprotease from placental cDNA libraries with homology to PAPP-A. This protein, which cleaves IGFBP-5 (and IGFBP-3), was named PAPP-A2 (83) and is present in human pregnancy serum where it releases IGF1 from the IGF1:IGFBP-5 complexes (84). PAPP-A2 appears as a monomer of 200 kDa in non-reducing gel electrophoresis and in contrast to PAPP-A does not bind to proMBP or associate with cell surfaces. Cleavage of IGFBP-5 by PAPP-A2 is not IGF-dependent, but as IGFBP-5 has been reported to have both IGF-dependent and IGF-independent effects in hOB cultures (see below), PAPP-A2 activity may also have major relevance in bone cell physiology. In agreement with this, homozygous PAPP-A2 KO mice show decreased post-natal growth along with reduced body length (85). Similarly, conditional PAPP-A2 KO in osteoblasts decreased body mass and bone length, although other tissue sources of PAPP-A2 may be involved in appropriate post-natal growth (86). *PAPP-A2* may represent a quantitative trait locus regulating body shape in mice (87, 88). Recently, two separate families (of Palestinian and Spanish ancestry) were found to have two different inactivating PAPP-A2 mutations that result in growth retardation in homozygous children (89, 90). Further analysis of affected individuals indicated significant increases in IGF1 in ternary ALS complexes with reduced free serum IGF1. In addition, affected individuals showed moderate microcephaly, mild BMD effects, and thin long bones. This phenotype was presumably associated with the inability of mutant PAPP-A2 to proteolyse IGFBP-3 and IGFBP-5 substrates.

## Other IGFBs in Bone Metabolism

### Insulin-Like Growth Factor-Binding Protein-5

Insulin-like growth factor-binding protein-5 is also present at high concentrations in bone matrix and has been associated with both inhibitory and stimulatory activities in bone cells and tissues. IGFBP-5 was reported to have IGF-dependent and IGF-independent effects in bone tissue, although the literature is conflicted in this area. IGFBP-5 was shown to enhance IGF-stimulated mitogenesis in hOB cultures (91, 92) and to stimulate the differentiation of two osteoblast cell lines in an IGF-independent fashion (93, 94). In ovariectomized rats, daily subcutaneous injection of IGFBP-5 increased osteoblast proliferation (95) and enhanced the association of IGF1 with bone cells possibly *via* specific cell-surface binding sites for IGFBP-5 (26, 96) or through a specific IGFBP-5 receptor on osteoblast membranes (97, 98). Disappointingly, however, a specific IGFBP-5 receptor has not been isolated or characterized further. Signaling studies suggest that the actions of IGFBP-5 in osteoblasts involve Ras association family isoform C activation of Erk-1/2 (19). The association of IGFBP-5 with four and a half LIM domain protein within the nucleus of U2 osteosarcoma cells has also been reported although the functional significance of this observation remains unknown (99). Although all the abovementioned findings are consistent with a stimulatory role for IGFBP-5 action in bone tissue (IGF-dependent or independent), some authors have reported contrary findings. For example, IGFBP-5 was reported to inhibit IGF1-stimulated proliferation in the U2 human osteosarcoma cell line (100), and transgenic mice overexpressing IGFBP-5 from the osteocalcin promoter showed decreased trabecular bone formation and reduced rates of mineral deposition during the first few weeks of post-natal life (15). Stromal cells isolated from transgenic animals also showed decreased levels of osteogenic markers. Constitutive overexpression of IGFBP-5 in the mouse osteoblast precursor cell line MC3T3-E1 also decreased osteogenic marker expression and delayed formation of mineralized nodules under osteogenic culture conditions (16). Finally, addition of exogenous wtIGFBP-5 or overexpression of IGFBP-5 from an adenovirus promoter inhibited osteoblast differentiation and growth of mouse metatarsal bones in short-term culture (20).

Although IGFBP-5 is cleaved in an IGF-independent manner by PAPP-A and PAPP-A2 (see above), it is also a substrate for other proteolytic enzymes. Matrix metalloproteinase-1 and -2 (MMP-1 and MMP-2) were shown to degrade IGFBP-5 in a time-dependent fashion in medium conditioned by the mouse MC-3T3-E1 cell line (101), and the complement component C1s was identified as an IGFBP5-specific protease in human dermal fibroblast-conditioned media (102). Following on from this, Mohan et al. described a disintegrin and metalloprotease-9 as an IGFBP-5 protease expressed in the U2 human osteosarcoma cell line (103). Although the importance of IGFBP-5 cleavage may (as for IGFBP4) lie with the regulation of free pericellular IGF concentrations, this is somewhat complicated by the observations of IGF-independent actions of IGFBP-5 described earlier. Clearly, these may also be impacted by IGFBP-5 cleavage. Further work is required to establish the role of IGFBP-5 in osteoblast differentiation and in bone tissue metabolism in general.

Finally, there are reports of broad-spectrum proteolytic enzyme families, which degrade IGFBP-5 (and other IGFBPs). These include plasmin (104), thrombin (105), the serine proteases cathepsin G, and elastase (106), although questions of specificity and biological relevance related to these proteases remain largely unanswered.

### Insulin-Like Growth Factor-Binding Protein-2

The literature describes both IGF-dependent and IGF-independent effects of IGFBP-2 in osteoblast cultures and bone tissues. An early study using a unilateral disuse osteoporosis model in the rat showed that osmotic minipump delivery of IGF2/IGFBP-2 complexes prevented the decrease in BMD in affected femurs associated with this model (107). A subsequent report from the same group showed that IGF2/IGFBP-2 complexes bound to heparin-Sepharose and that it was suggested that such complexes may associate with ECM components in bone tissue potentially increasing the local concentration of IGFs (108). In agreement with the abovementioned findings, IGFBP-2 potentiated IGF2-induced increases in ALP activity in cultures of rat tibial osteoblasts (109), and we have demonstrated the same effect of IGFBP-2 on IGF1-stimulated ALP activity in differentiating human dental pulp cells (110). Studies in IGFBP-2 KO mice indicated gender-specific differences in osteogenic phenotype with increased cortical thickness and periosteal circumference in female mice but reduced cortical bone area and trabecular volume in male KOs (111). Although difficult to rationalize, it clearly suggests interplay between the IGF axis and other hormone systems. This same group also reported impaired osteoclastogenesis in bone marrow cells derived from *igfbp2−/− mice* and a transfection study in these cells and indicated that both the IGF and heparin-binding domain (HBD) of IGFBP-2 were required for osteoclast generation (112). This description of IGF-independent effects of IGFBP-2 *in vitro* was confirmed in concurrent studies demonstrating restoration of osteogenic phenotype in *igfbp2−/−* bone marrow cells by addition of a HBD peptide derived from IGFBP-2. In addition, *in vivo* administration of HBD peptide restored osteoblast number in *igfbp2−/−* mice (17). Recently, studies in the mouse MC-3T3 pre-osteoblast cell line showed that IGFBP-2 can bind and inhibit the activity of receptor phosphotyrosine phosphatase β causing increased levels of phosphorylated PTEN, activation of Akt, and stimulation of osteogenesis (17, 18). Further reports from this laboratory highlight the importance of the scaffold/adaptor protein IRS-1, PKCζ, and early activation of AMP-dependent protein kinase in the osteoblast differentiation of primary rat calvarial cells and the differentiating MC-3T3 cell line (21, 22). It should be noted that IGFBP-2 is also a PAPP-A substrate, although this IGFBP is cleaved less efficiently than IGFBP-4 and IGFBP-5 (113).

## IGFBP-1, -3, and -6

Although IGFBP-1, -3, and -6 have all been reported to be expressed in osteoblasts and to be present in bone tissue (114), there are fewer data describing the functions of these three IGFBPs. IGFBP-1 was expressed at low levels in primary hOB cultures under regulation of glucocorticoid and insulin although the physiological relevance of this effect in bone tissue has not been established (115). A recent prospective study (10-year follow-up) in a cohort of elderly women reported a positive correlation between serum IGFBP-1 and osteoporotic fracture, suggesting an IGF-independent osteopenic effect of IGFBP-1 (116). Further data are required on IGFBP-1 and its effects (if any) on bone physiology.

A very early study reported inhibition of IGF1-stimulated DNA synthesis in two osteoblast cell lines by intact IGFBP-3 (117). This inhibitory effect on both IGF1- and IGF2-stimulated DNA synthesis was confirmed in cultures of rat calvarial cells (118). Although these data suggest an inhibitory role for IGFBP-3 in bone metabolism, other *in vivo* data (119) and cross-sectional studies in a cohort of female patients with postmenopausal osteoporosis suggest an anabolic role for IGFBP-3 in maintaining bone density (120).

Insulin-like growth factor-binding protein--6 mRNA was expressed in primary osteoblast cultures derived from fetal rat calvaria (121), and the expression of both mRNA and protein was upregulated in a dose-dependent fashion by cortisol or retinoic acid treatment (122, 123) cultures. Conversely, IGFBP-6 expression was negatively regulated by TGFβ1 in the same cell culture system (124). IGFBP-6 shows a higher affinity for IGF2 than IGF1. Accordingly, it was shown to be a more potent inhibitor of IGF2-stimulated DNA and glycogen synthesis in hOB cells than IGF1 (125). This inhibitory effect of IGFBP-6 was confirmed in the SaoS2 human osteosarcoma cell line using a stable antisense transfection strategy to demonstrate that the anti-differentiative activity of all-trans retinoic acid (Vitamin D) was at least partly mediated *via* IGFBP-6 (126). More recently, IGFBP-6 has been shown to interact with the thyroid hormone receptor alpha1 and to inhibit the tri-iodothyronine-induced increase in osteoblast marker expression in the human U2-OS osteosarcoma cell line (127). In contrast to these reports, the inhibitory effect of IGFBP-6 attenuated by intracellular interaction with the LIM mineralizing protein in both human and mouse osteoblastic cells (128), and one study reported a stimulatory effect of IGFBP-6 on DNA synthesis and mitogenesis in the human osteosarcoma Saos-2/B-10 cell line (129). As for IGFBP-1 and IGFBP-3, the role of IGFBP-6 in osteogenesis and bone tissue physiology has been underreported, and further studies are required to elucidate the role of these 3 IGFBPs in osteogenesis and bone physiology.

## Conclusion

Six decades have passed since the initial description of the anabolic role of IGF1 in skeletal tissue (130). In the intervening years, much progress has been made in defining the actions of IGF1 and IGF2 and other members of the IGF axis in bone tissue at all stages of development. This review has focused specifically on the function of IGFBPs in osteogenic tissues – both IGF-dependent and IGF-independent. However, the IGF axis acts in a co-ordinated fashion and is integrated with other hormonal systems and growth factor axes to regulate skeletal tissue development and maintenance. It is anticipated that in this and other aspects of IGF axis physiology, many important observations will be made in the near future.

## Author Contributions

All the authors contributed to the writing and editing of the manuscript.

## Conflict of Interest Statement

The authors declare that the research was conducted in the absence of any commercial or financial relationships that could be construed as a potential conflict of interest.

## Abbreviations

IGF, insulin-like growth factor; IGFBP, IGF-binding proteins; IGF1R, IGF1 receptor; hDPC, human dental pulp cells; PAPP-A, pregnancy-associated plasma protein-A; STC, stanniocalcin; BMD, bone mineral density; hOB, human osteoblasts.
