# Corrigendum: Insulin- like Growth Factor-Binding Protein Action in Bone Tissue: A Key Role for Pregnancy- Associated Plasma Protein-A

**DOI:** 10.3389/fendo.2018.00510

**Published:** 2018-09-04

**Authors:** James Beattie, Hasanain Al-Khafaji, Pernille R. Noer, Hanaa Esa Alkharobi, Aishah Alhodhodi, Josephine Meade, Reem El-Gendy, Claus Oxvig

**Affiliations:** ^1^Division of Oral Biology, Leeds School of Dentistry, Level 7 Wellcome Trust Brenner Building, University of Leeds, St James University Hospital, Leeds, United Kingdom; ^2^Department of Molecular Biology and Genetics, Aarhus University, Aarhus, Denmark; ^3^Department of Oral Biology, Dental College, King AbdulAziz University, Jeddah, Saudi Arabia; ^4^Department of Oral Pathology, Faculty of Dentistry, Suez Canal University, Ismailia, Egypt

**Keywords:** insulin-like growth factor-binding protein-4, bone, pregnancy-associated plasma protein-A, proteolysis, insulin-like growth factor-binding protein-5

In the original article, we neglected to include the funders. HA-K acknowledges the Higher Education Committee for Education and Development (HCED), Office of Prime Minister, Iraq for financial support. AA and HA acknowledge the Royal Embassy of Saudi Arabia – Cultural Bureau (UK) for financial support. RE-G acknowledges WELMEC, a Centre of Excellence in Medical Engineering funded by the Wellcome Trust and EPSRC, under grant number WT 088908/Z/09/Z for financial support.

The authors apologize for this error and state that this does not change the scientific conclusions of the article in any way.

The original article has been updated.

## Conflict of interest statement

The authors declare that the research was conducted in the absence of any commercial or financial relationships that could be construed as a potential conflict of interest.

